
JOURNAL INFORMATION
==============================
NLM Title Abbreviation: CPT Pharmacometrics Syst Pharmacol
Journal ID: CPT Pharmacometrics Syst Pharmacol
NLM Journal ID: 101580011
Journal ID: PSP4

CPT: Pharmacometrics & Systems Pharmacology

EISSN: 2163-8306
Publisher: Wiley

ARTICLE INFORMATION
==============================
PMCID: PMC11474673
DOI: 10.1002/psp4.12824
Article ID: PSP412824
Article version: 1
Subjects: Issue Information, Issue Information

Issue Information

Electronic publication date: 2023 Sep 19
Publication date: 2023 Sep
Volume: 12
Issue: 9
First page: 1183
Last page: 1186
Copyright: © 2023 The Authors. CPT: Pharmacometrics & Systems Pharmacology published by Wiley Periodicals LLC on behalf of American Society for Clinical Pharmacology and Therapeutics.
License: This is an open access article under the terms of the http://creativecommons.org/licenses/by/4.0/ License, which permits use, distribution and reproduction in any medium, provided the original work is properly cited.
License URL: https://creativecommons.org/licenses/by/4.0/

Figure count: 0
Table count: 0
Page count: 3

source-schema-version-number: 2.0
cover-date: September 2023
details-of-publishers-convertor: Converter:WILEY_ML3GV2_TO_JATSPMC version:6.4.9 mode:remove_FC converted:15.10.2024

==============================

An Official Journal of ASCPT and ISoP

### CPT: Pharmacometrics & Systems Pharmacology

Open Access

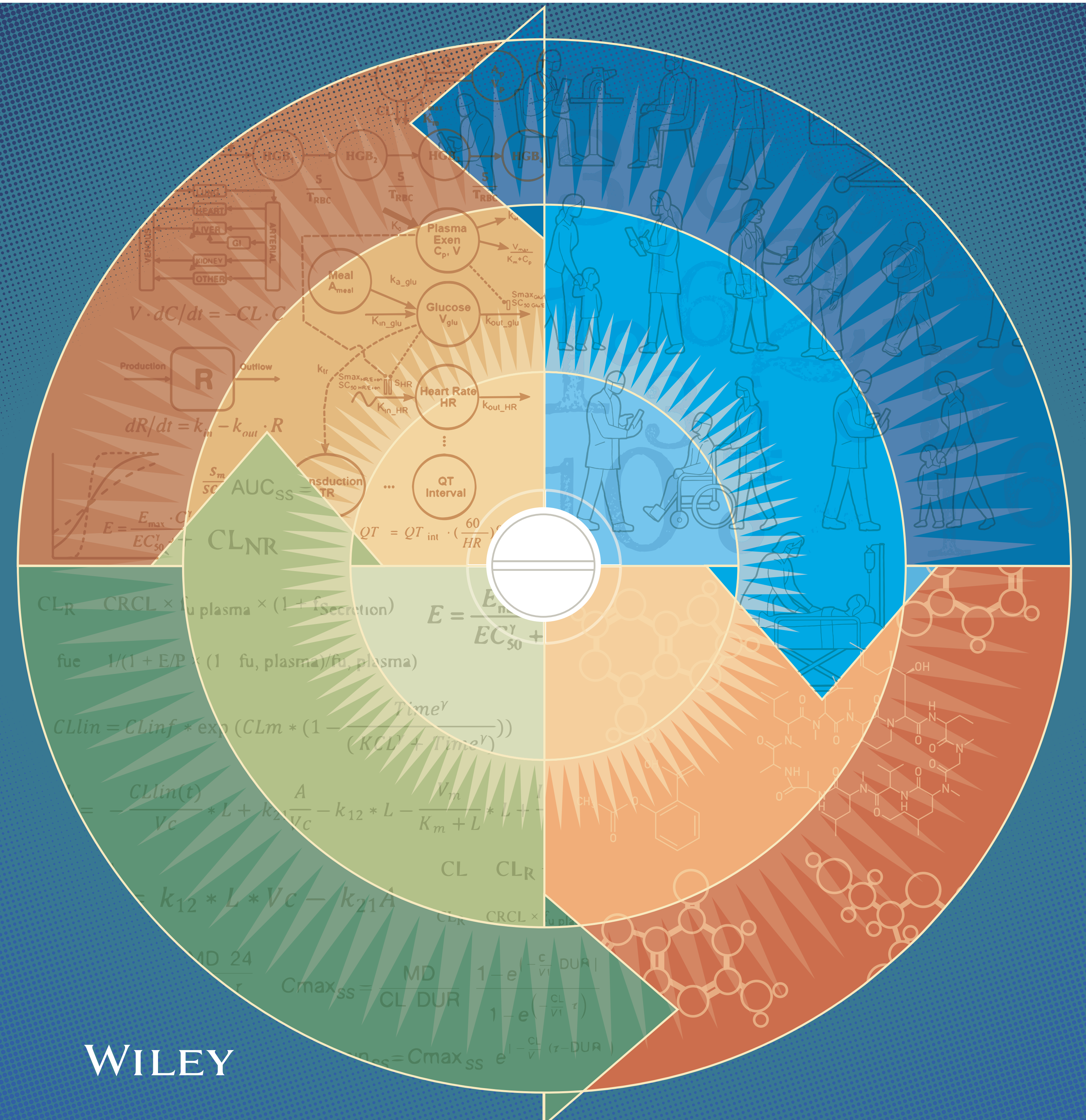

WILEY

### CPT: Pharmacometrics & Systems Pharmacology

WILEY

### Editor-in-Chief

France Mentré, MD, PhD, *School of Medicine of University Paris Diderot, Paris, France*

### Deputy Editor-in-Chief

Karen Rowland Yeo, PhD, *Certara UK Ltd., Sheffield, UK*

### Associate Editors

Paolo Denti, PhD, *University of Cape Town, Cape Town, South Africa*
Jonathan French, ScD, *Metrum Research Group, Tariffville, CT, USA*
Stefanie Hennig, MSc, PhD, *Certara Strategic Consulting, Brisbane, Australia*
Andrew Hooker, PhD, *Uppsala University, Uppsala, Sweden*
Sriram Krishnaswami, PhD, *Pfizer, Groton, CT, USA*
Vikram Sinha, PhD, *Novartis Institute of Biomedical Research, Berwyn, PA, USA*
Ping Zhao, PhD, *Bill & Melinda Gates Foundation, Seattle, WA, USA*
Eric Sobie, PhD, *Metrum Research Group, Tariffville, CT, USA*

### Editors-in-Training

Rajat Desikan, PhD, *GSK, UK, Middlesex, UK*
Priya Jayachandran, PharmD, *Regeneron Pharmaceuticals, Inc., San Francisco, CA, USA*

### American Society for Clinical Pharmacology and Therapeutics

***Chief Executive Officer***
Abigail W. Gorman, *Alexandria, VA*
***Chief Operating Officer***
Elise S. Laffman-Johnson, *Alexandria, VA*
***Managing Editor***
Alethea B. Gerding, *Alexandria, VA*
***Deputy Managing Editor***
Alaina Webster, *Alexandria, VA*
***Senior Editorial Coordinator***
Aimee Greene, *Alexandria, VA*

### Editorial Board

Vivaswath S. Ayyar, PhD, *Janssen Research & Development, Spring House, PA*
Robert Bies, PharmD, PhD, *State University of New York at Buffalo, Buffalo, NY, USA*
Marc Birtwistle, PhD, *Clemson University Clemson, SC, USA*
Marylore Chenel, PharmD, PhD, *Pharmetheus Uppsala, Sweden*
Emmanuel Chigutsa, PhD, *Eli Lilly and Company, Indianapolis, IN, USA*
Teresa Dalla Costa, PhD, *Federal University of Rio Grande do Sul - UFRGS Porto Alegre, Brazil*
Paolo Denti, PhD, *University of Cape Town Cape Town, South Africa*
Narendra M Dixit, PhD, *Indian Institute of Science, Bengaluru, India*
Anne-Gaelle Dosne, PharmD, PhD, *Janssen Research & Development, Beerse, Belgium*
James M. Gallo, PharmD, PhD, *University at Buffalo, Buffalo, NY, USA*
Wei Gao, PhD, *EMD Serono, Billerica, MA, USA*
Manish Gupta, PhD, FCP, *GenMab; Princeton, NJ, USA*
Bart Hendriks, PhD, *Novartis Institutes for BioMedical Research Cambridge, MA, USA*
Nick Holford, MBChB, *The University of Auckland Auckland, New Zealand*
Brett A. Howell, PhD, *DILIsym Services Division, Simulations Plus, Inc., Research Triangle Park, NC, USA*
Manuel Ibarra, PhD, *Universidad de la República, Montevideo, Uruguay*
Siv Jönsson, PhD, *Uppsala University Sweden, Uppsala, Sweden*
Kristin Karlsson, PhD, *Uppsala University Uppsala, Sweden*
Helen Kastrissios, PhD, *QuanTx Consulting, Brisbane, Australia*
Ron Keizer, PhD, *InsightRX San Francisco, CA, USA*
Steven E. Kern, PhD, *Global Health Labs, Bellevue, WA, USA*
Andrzej Kierzek, PhD, *Certara QSP; Sheffield, United Kingdom*
Qi Liu, PhD, *US Food and Drug Administration, Silver Spring, MD*
James Lu, PhD, *Genentech, Inc., South San Francisco, CA, USA*
Rajanikanth (Raj) Madabushi, PhD, *US Food and Drug Administration, Silver Spring, MD*
Efthymios Manolis, MSc, *European Medicines Agency London, United Kingdom*
Samer Mouksassi, PharmD, PhD, *Certara, Cairo, Egypt*
Jackson K. Mukonzo, PhD, *Makerere University, Kampala Uganda*
Joakim Nyberg, PhD, *Pharmetheus, Uppsala, Sweden*
Elsje Pienaar, PhD, *Weldon School of Biomedical Engineering, Purdue University, West Lafayette, IN, USA*
Daniel Scholes Rosenbloom, PhD, *Merck & Co., Rahway, NJ, USA*
Joe Standing, PhD, *University College London London, United Kingdom*
Natasha Strydom, PhD, *Amgen, Inc., San Francisco, CA, USA*
Yuichi Sugiyama, PhD, *Riken Innovation Center Sugiyama Laboratory Yokohama, Japan*
Joel Tarning, PhD, *Mahidol-Oxford Tropical Medicine Research Unit Bangkok, Thailand*
Nadia Terranova, PhD, *Merck KGaA, Lausanne, Switzerland*
Michael Tortorici, PharmD, PhD, *Aro Biotherapeutics, Philadelphia, PA, USA*
Coen van Hasselt, PhD, *Leiden University, Leiden, the Netherlands*
Paolo Vicini, PhD, MBA, *Confo Therapeutics, Ghent, Belgium*
Camille Vong, PhD, *Novartis AG Pharma, Basel, Switzerland*
Xinyuan (Susie) Zhang, PhD, *Daiichi Sankyo, Inc. Basking Ridge, NJ, USA*

### Overview

*CPT: Pharmacometrics & Systems Pharmacology* (PSP) is an official open access journal of the American Society for Clinical Pharmacology and Therapeutics (ASCPT) and International Society of Pharmacometrics (ISoP).

*CPT: Pharmacometrics & Systems Pharmacology* is a cross-disciplinary journal devoted to publishing advances in quantitative (e.g., modeling and simulation) methods as applied in pharmacology, physiology and therapeutics in humans. The journal welcomes original research articles, reviews and tutorials that bridge the following areas: pharmacometrics, modeling and simulation as applied to the design and evaluation of clinical trials, systems pharmacology modeling, particularly with a mechanistic link to human (patho)physiology, disease modeling, "population" or mixed-effects pharmacokinetics and pharmacodynamics (PKPD) modeling, modeling and simulation to support translational research, physiologically-based pharmacokinetics (PBPK), model-based meta-analyses of clinical trials, mechanism-based pharmacokinetic-pharmacodynamic modeling, computational pharmacology, bioinformatics, comparative efficacy, effectiveness and cost-effectiveness. Systems pharmacology may involve the application of systems biology approaches to study drug activities, targets and effects. The discipline is often defined with reference to engineering and pharmacological principles as the quantitative analysis of the dynamic interactions between drugs and a biologic system that aims to understand the behavior of the system as a whole. The common focus will be on quantitative methods that improve our understanding of pharmacology and therapeutics in humans.

**Abstracting and Indexing**
- Embase (Elsevier)
- SCOPUS (Elsevier)
- MEDLINE/PubMed indexing

**Society Information**

The American Society for Clinical Pharmacology and Therapeutics (ASCPT) was founded in 1900, and consists of over 2,200 professionals whose primary interest is to advance the science and practice of clinical pharmacology and translational medicine for the therapeutic benefit of patients and society. ASCPT is the largest scientific and professional organization serving the disciplines of Clinical Pharmacology and Translational Medicine.

**All articles published in this journal are freely available to read, download, and share. Articles are published under the terms of the Creative Commons Attribution Non-Commercial License which permits use, distribution, and reproduction in any medium, provided the original work is properly cited and is not used for commercial purposes.**

**Disclaimer**

The Publisher, the American Society for Clinical Pharmacology and Therapeutics, the International Society of Pharmacometrics, and Editors cannot be held responsible for errors or any consequences arising from the use of information contained in this journal; the views and opinions expressed do not necessarily reflect those of the Publisher, the American Society for Clinical Pharmacology and Therapeutics, the International Society of Pharmacometrics or Editors, neither does the publication of advertisements constitute any endorsement by the Publisher, the American Society for Clinical Pharmacology and Therapeutics, the International Society of Pharmacometrics or Editors of the products advertised.

**Publisher**

*CPT: Pharmacometrics & Systems Pharmacology* is published by Wiley Periodicals, LLC, 101 Station Landing, Suite 300, Medford, MA 02155; Telephone: 781-388-8200; Fax: 781-388-8210.

CONTENTS

CPT: Pharmacometrics & Systems Pharmacology

### TUTORIAL

### TUTORIAL

1187 Pharmacokinetic analysis across studies to drive knowledge-integration: A tutorial on individual patient data meta-analysis (IPDMA)
**Rob C. van Wijk, Marjorie Z. Imperial, Radojka M. Savic and Belén P. Solans**

### RESEARCH

### ARTICLES

1201 A systematic approach towards missing lab data in electronic health records: A case study in non-small cell lung cancer and multiple myeloma
**Arjun Sondhi, Janick Weberpals, Prakirthi Yerram, Chengsheng Jiang, Michael Taylor, Meghna Samant and Sarah Cherng**

1213 Development of a population pharmacokinetic model to characterize the pharmacokinetics of intrathecally administered tominersen in cerebrospinal fluid and plasma
**Yumi Yamamoto, Patricia Sanwald Ducray, Marcus Björnsson, Kevin Smart, Paul Grimsey, Suresh Vatakuti, Agnes Portron, Benoit Massonnet, Daniel A. Norris and Hanna E. Silber Baumann**

1227 Model-based prediction of progression-free survival for combination therapies in oncology
**Marcus Baaz, Tim Cardilin and Mats Jirstrand**

1238 Saliva as a noninvasive sampling matrix for therapeutic drug monitoring of intravenous busulfan in Chinese patients undergoing hematopoietic stem cell transplantation: A prospective population pharmacokinetic and simulation study
**Baohua Xu, Ting Yang, Jianxing Zhou, You Zheng, Jingting Wang, Qingxia Liu, Dandan Li, Yifan Zhang, Maobai Liu and Xuemei Wu**

1250 Seasonal influence on respiratory tract infection severity including COVID-19 quantified through Markov Chain modeling
**Rob C. van Wijk, Laurynas Mockeliunas, Caryn M. Upton, Jonathan Peter, Andreas H. Diacon and Ulrika S. H. Simonsson**

1262 Exposure-safety and exposure-efficacy analyses for tisotumab vedotin for patients with locally advanced or metastatic solid tumors
**Chaitali Passey, Jenna Voellinger, Leonid Gibiansky, Rudy Gunawan, Leonardo Nicacio, Ibrahima Soumaoro, William D. Hanley, Helen Winter and Manish Gupta**

1274 A physiologically-based pharmacokinetic model for tuberculosis drug disposition at extrapulmonary sites
**Aparna Ramachandran and Chetan J. Gadgil**

1285 Development of a multiscale mechanistic modeling framework integrating differential cellular kinetics of CAR T-cell subsets and immunophenotypes in cancer patients
**Ahmed M. Salem, Ganesh M. Mugundu and Aman P. Singh**

1305 Assessment of ibrutinib scheduling on leukocyte, lymph node size and blood pressure dynamics in chronic lymphocytic leukemia through pharmacokinetic-pharmacodynamic models
**Eman I. K. Ibrahim, Mats O. Karlsson and Lena E. Friberg**

1319 Semimechanistic pharmacokinetic–pharmacodynamic model of tripegfilgrastim for pediatric patients after chemotherapy
**Soyoung Lee, Kyung Taek Hong, In-Jin Jang, Kyung-Sang Yu, Hyoung Jin Kang and Jaeseong Oh**

1335 Development and application of a PBPK modeling strategy to support antimalarial drug development
**Nada Abla, Eleanor Howgate, Karen Rowland-Yeo, Maurice Dickins, Mackenzie C. Bergagnini-Kolev, Kuan-Fu Chen, Savannah McFeely, Jennifer J. Bonner, Laura G. A. Santos, Nathalie Gobeau, Howard Burt, Zoe Barter, Hannah M. Jones, David Wesche, Susan A. Charman, Jörg J. Möhrle, Jeremy N. Burrows and Lisa M. Almond**

1347 Can a propensity score matching method be applied to assessing efficacy from single-arm proof-of-concept trials in oncology?
**Shu-Wen Teng, Yu-Cheng Su, Ravikumar Pallantla, Madhav Channavazzala, Rukmini Kumar, Yucheng Sheng, Hao Wang, Crystal Wang and Archie Tse**

1358 Prediction of relative change in free nerve growth factor following subcutaneous administration of tanezumab, a novel monoclonal antibody to nerve growth factor
**Satoshi Shoji, Akiyuki Suzuki, Parya Nouri, Chun-Hua Cai, Puneet Gaitonde and Scott Marshall**
